# Radiogenomics analysis reveals the associations of dynamic contrast-enhanced–MRI features with gene expression characteristics, PAM50 subtypes, and prognosis of breast cancer

**DOI:** 10.3389/fonc.2022.943326

**Published:** 2022-07-28

**Authors:** Wenlong Ming, Yanhui Zhu, Yunfei Bai, Wanjun Gu, Fuyu Li, Zixi Hu, Tiansong Xia, Zuolei Dai, Xiafei Yu, Huamei Li, Yu Gu, Shaoxun Yuan, Rongxin Zhang, Haitao Li, Wenyong Zhu, Jianing Ding, Xiao Sun, Yun Liu, Hongde Liu, Xiaoan Liu

**Affiliations:** ^1^ State Key Laboratory of Bioelectronics, School of Biological Science and Medical Engineering, Southeast University, Nanjing, China; ^2^ Department of Breast Surgery, The First Affiliated Hospital of Nanjing Medical University, Nanjing, China; ^3^ Collaborative Innovation Center of Jiangsu Province of Cancer Prevention and Treatment of Chinese Medicine, School of Artificial Intelligence and Information Technology, Nanjing University of Chinese Medicine, Nanjing, China; ^4^ Department of Information, The First Affiliated Hospital of Nanjing Medical University, Nanjing, China

**Keywords:** breast cancer, radiogenomics, radiomics, PAM50 subtypes, DCE-MRI, machine learning

## Abstract

**Background:**

To investigate reliable associations between dynamic contrast-enhanced magnetic resonance imaging (DCE-MRI) features and gene expression characteristics in breast cancer (BC) and to develop and validate classifiers for predicting PAM50 subtypes and prognosis from DCE-MRI non-invasively.

**Methods:**

Two radiogenomics cohorts with paired DCE-MRI and RNA-sequencing (RNA-seq) data were collected from local and public databases and divided into discovery (*n* = 174) and validation cohorts (*n* = 72). Six external datasets (*n* = 1,443) were used for prognostic validation. Spatial–temporal features of DCE-MRI were extracted, normalized properly, and associated with gene expression to identify the imaging features that can indicate subtypes and prognosis.

**Results:**

Expression of genes including RBP4, MYBL2, and LINC00993 correlated significantly with DCE-MRI features (q-value < 0.05). Importantly, genes in the cell cycle pathway exhibited a significant association with imaging features (*p*-value < 0.001). With eight imaging-associated genes (*CHEK1*, *TTK*, *CDC45*, *BUB1B*, *PLK1*, *E2F1*, *CDC20*, and *CDC25A*), we developed a radiogenomics prognostic signature that can distinguish BC outcomes in multiple datasets well. High expression of the signature indicated a poor prognosis (*p*-values < 0.01). Based on DCE-MRI features, we established classifiers to predict BC clinical receptors, PAM50 subtypes, and prognostic gene sets. The imaging-based machine learning classifiers performed well in the independent dataset (areas under the receiver operating characteristic curve (AUCs) of 0.8361, 0.809, 0.7742, and 0.7277 for estrogen receptor (ER), human epidermal growth factor receptor 2 (HER2)-enriched, basal-like, and obtained radiogenomics signature). Furthermore, we developed a prognostic model directly using DCE-MRI features (*p*-value < 0.0001).

**Conclusions:**

Our results identified the DCE-MRI features that are robust and associated with the gene expression in BC and displayed the possibility of using the features to predict clinical receptors and PAM50 subtypes and to indicate BC prognosis.

## Introduction

Breast cancer (BC) remains a leading death cause in women and exhibits high heterogeneity in both clinical and molecular (gene expression/mutation) respects (1, 2). According to gene expression, BC is defined as five intrinsic molecular subtypes, namely, luminal-A, luminal-B, human epidermal growth factor receptor 2 (HER2)-enriched, basal-like, and normal-like (3). Clinically, BC is routinely divided into four subtypes based on the expression of four histopathological receptors (estrogen receptor (ER), progesterone receptor (PR), HER2, and Ki-67) (4). For the subtypes, the diagnosis, treatment, prognosis, and gene expression are very different. Therefore, grasping and monitoring the molecular characteristics and gene expression patterns timely and accurately are meaningful for diagnosis, subtyping, and prognosis of BC.

Medical imaging is one kind of non-invasive approach for characterizing the disease. By extracting high-throughput quantitative imaging features from medical images and applying the information to clinical-decision support systems, radiomics is gaining more attention in cancer research (5, 6). However, artificial intelligence (AI) is developing rapidly, and some models were presented to help the automatic segmentation or computer-aided diagnosis from clinical cancer imaging (7–11). For example, by using AI and radiomics, researchers can assess the personalized cancer risk in the early breast magnetic resonance imaging (MRI) exams and can discriminate benign or malignant breast lesions automatically (10, 11). Radiogenomics is an extended field of radiomics, which aims to identify the association between medical imaging features and genetic characteristics or gene expression in the concept of precision medicine (12). It is helpful to study molecular characteristics directly from imaging features to establish typing, diagnosis, and prognosis for clinical applications (12, 13). Among different imaging techniques, dynamic contrast-enhanced magnetic resonance imaging (DCE-MRI) is widely used in BC research for its strengths of three-dimensional resolution and high imaging quality (12, 14).

Previous studies suggested that the DCE-MRI features were related to the gene expression level of both coding RNAs and non-coding RNAs and can reflect the dysregulation of disease-related gene pathways in BC patients (15–24). For example, more irregular and larger tumors usually correlated with higher expression of genes of cell cycle and DNA damage checkpoint (19), and the associations of miRNAs with imaging features differed across BC subtypes (21). DCE-MRI features were found to be associated with the deregulation or genetic alterations of some important pathways such as the mTOR pathway and oncogenic signaling pathways (22, 23). Some works also attempted to establish prediction models for clinical biomarkers (such as ER and PR) as well as immunohistochemistry (IHC) subtypes of BC based on quantitative imaging features with a machine learning or deep learning approach (25–33). A study combined the MRI features from both peritumoral and intratumoral regions to predict the HER2-enriched molecular subtype and achieve an area under the curve (AUC) of 0.89 (31). In a recent large meta-analysis, the IHC subtypes of BC were predicted non-invasively by the radiomics analysis based on MRI features (33). In addition, uncovering the ability of imaging features to assess the treatment response and predict clinical outcomes in BC is a valuable research aspect. Some radiomics signatures on MRI were developed to predict the clinical outcomes of BC patients such as the metastasis of axillary lymph nodes and disease-free survival (DFS) (27, 34–37).

Despite the advancement in this field, most studies of association analysis were only on a single-central dataset, which might lead to the systematic bias of data, especially for the medical imaging data (38). Although many works have been performed to establish the prediction models for BC biomarkers or IHC subtypes, very few studies established and validated the PAM50 subtypes classifiers. It is very critical to improve the identification accuracy of the PAM50 subtypes in clinical diagnosis, since the currently widely used IHC-based alternative subtyping uses the expression levels of only four IHC markers (ER, PR, HER2, and Ki67), whereas the PAM50 subtyping system is able to portray the typical and comprehensive transcriptomic characteristics of BC.

In this work, we collected DCE-MRI and RNA-sequencing (RNA-seq) data from two cohorts consisting of multiple centers with over 300 BC samples. With the datasets, we performed a comprehensive analysis by extracting and sorting quantitative DCE-MRI features and associating the imaging features to gene expression to explore the possibility of constructing models to predict prognosis and subtyping for BC. We revealed a similar pattern of association in the two cohorts and provided a picture of the relationship between gene expression, imaging features, and BC prognosis. In addition, we established and validated the prediction models for each PAM50 intrinsic molecular subtype based on quantitative DCE-MRI features for the first time.

## Materials and methods

### Patient selection and pathological review

The discovery cohort comprised female patients who were histologically confirmed to have invasive ductal carcinoma between August 2016 and December 2018. Both the preoperative T1-weighted DCE-MRI data and matched tumor tissue specimens can be accessible to the patients from the institutional database. The inclusion and exclusion criteria of samples are shown in **Figure S1**. The final discovery cohort was composed of 174 cases. A multi-institutional dataset (TCGA-BRCA, *n* = 1,090) was retrieved from The Cancer Imaging Archive (TCIA) database and The Cancer Genome Atlas (TCGA), and 72 cases were included in the validation cohort whose DCE-MRI was acquired on a 1.5-Tesla magnet strength by GE scanners. We further retrieved six datasets (*n* = 1,443) from the Gene Expression Omnibus (GEO) database to assess prognosis, with the series accession numbers GSE1456, GSE3494, GSE7390, GSE20685, GSE25055, and GSE25065.

The detailed clinical characterization of the two cohorts is listed in **Table 1**. ER, PR, HER2, and Ki67 were used to determine the clinical IHC subtypes for each patient in the discovery cohort. ER-positive, HER2-negative, high PR expression (more than 20%), and low Ki67 expression (less than 20%) samples were defined as luminal-A. ER-positive, HER2-negative, low PR expression, or high Ki-67 expression samples were defined as luminal-B. Furthermore, ER- and HER2-positive samples were defined as luminal-B as well. ER-negative, PR-negative, and HER2-positive samples were HER2-positive, and finally, all negative samples were triple-negative BC (TNBC). Two-sided Fisher’s exact test or Pearson’s chi-squared test was used to assess differences in the clinical or transcriptomic characteristics of BC samples in the discovery and validation cohorts.

**Table 1:** The clinical and transcriptomic characteristics in two cohorts.

Characteristics	The discovery cohort (*n* = 174)	The validation cohort (*n* = 72)	*p*-Value
Age	≤50 years: 95 (54.6)/>50 years: 79 (45.4)	≤50 years: 30 (41.7)/>50 years: 42 (58.3)	0.088^a^
IHC ER status PR status HER2 status Ki67 status	P: 127 (73.0)/N: 47 (27.0) P: 111 (63.8)/N: 63 (36.2) P: 36 (20.7)/N: 138 (79.3) High: 136 (78.2)/low: 38 (21.8)	P: 61 (84.7)/N: 11 (15.3) P: 55 (76.4)/N: 17 (23.6) P: 14 (19.4)/N: 37 (51.4)/NA: 21 (29.2) NA	0.071^a^ 0.077^a^ 0.407^a^ NA
IHC-based subtypes Luminal-A Luminal-B HER2-positive Triple-negative	28 (16.1) 101 (58.1) 15 (8.6) 30 (17.2)	NA NA NA NA	NA NA NA NA
PAM50 intrinsic subtypes Luminal-A Luminal-B HER2-enriched Basal-like Normal-like	49 (28.1) 43 (24.7) 29 (16.7) 43 (24.7) 10 (5.8)	44 (61.1) 9 (12.5) 5 (6.9) 10 (13.9) 4 (5.6)	<0.001^b^
Prognostic risk MammaPrint Oncotype DX	High: 131 (75.3)/low: 43 (24.7) High: 168 (96.6)/intermediate or low: 6 (3.4)	High: 55 (76.4)/low: 17 (23.6) High: 67 (93.1)/intermediate or low: 5 (6.9)	0.984^a^ 0.385^a^
Pathological stage I stage II stage III stage	55 (31.6) 94 (54.0) 25 (14.4)	17 (23.6) 47 (65.3) 8 (11.1)	0.267^a^

Clinical, MRI, and molecular data for both cohorts were available. The patient distribution of the two cohorts was not different except for the PAM50 molecular subtypes. Unless otherwise indicated, data are number of samples or the p-value of statistical test, and the data in parentheses are percentages.

P, receptor status; N, negative; NA, not available; IHC, immunohistochemistry; ER, estrogen receptor; PR, progesterone receptor; HER2, human epidermal growth factor receptor 2.

a p-Value for the two-sided Pearson’s chi-squared test.

b p-Value for the two-sided Fisher’s exact test.

### Extraction of quantitative dynamic contrast-enhanced–mri features

For original imaging data, the first step of its application was to compute various quantitative features, which can reflect the different properties of the images. The detailed imaging protocols for the two cohorts are discussed subsequently. T1-weighted DCE-MR images in the discovery cohort were scanned in the axial position and performed by using a Siemens TrioTim 3-Tesla scanner (Siemens Healthcare, Erlangen, Germany). The parameters for the bilateral protocol of most images are as follows: repetition time, 423 ms; echo time, 15.7 ms; slice thickness, 0.9 mm; flip angle, 10°; field of view, 340 × 340 mm; and matrix size, 448 × 448 pixels. Gadolinium-diethylenetriamine pentaacetic acid (Gd-DTPA) in a dose of 0.1 mmol/kg was injected intravenously into the body at an amount of 15 ml. Three-dimensional dynamic sequences were performed with six time points, including one pre-contrast and five post-contrast (from approximately 1 min after contrast to approximately 4.5 min). MRI data from the validation cohort, including one pre-contrast and three to five contrast-enhanced images, were obtained by using a T1-weighted three-dimensional spoiled gradient-echo sequence with a gadolinium-based contrast agent. The in-plane resolution of images ranged from 0.53 to 0.86 mm, spacing between slices ranged from 2 to 3 mm, the flip angle was 10°, and the acquisition matrix was 256 × 192.

In extracting features for the collected and filtered imaging data, we first localized and segmented the tumor lesions by using the threshold segmentation method and manual correction by two radiologists. We applied threshold segmentation on each 3D image from the subtracted images of the first post-contrast sequences to generate the roughly 3D tumor masks using the open-source software 3D Slicer. Then a senior radiologist (WC, with 10 years of breast imaging experience) and a junior radiologist (YZ, with 3 years of breast imaging experience) manually corrected the tumor masks in 3D Slicer. The two radiologists were blinded to the clinical data and confirmed the corrected tumor masks in consensus. Sequences for DCE-MR images at four time points were selected and further analyzed in both cohorts, including pre-contrast, and early, middle, and late post-contrast (approximately 1, 3, and 4.5 min, respectively). To avoid data heterogeneity bias, the N4 bias correction algorithm was applied to remove shading artifacts in the 3T MR images (39). Next, a Python package pyradiomics (version 2.2.0) was used to image normalization and quantitative imaging features calculation (40). Image normalization was performed by remapping the histogram to fit within μ ± 3σ (μ, mean gray level within the volume of tumor segmentation; σ, gray-level standard deviation). After that, the images were resampled to an isotropic voxel resolution of 1 mm using the B-spline method before feature extraction. Image pre-processing and feature extraction were conducted in Python 3.5.2. Totally, 15,494 high-throughput quantitative imaging features were calculated for each case based on the basic imaging features provided by Image Biomarker Standardisation Initiative (IBSI). The details are described in Supplementary Methods of **Supplementary File S1**.

### Rna-sequencing and calculation of breast cancer transcriptomic characteristics

After imaging feature extraction, we first attempted to reveal the reliable association between quantitative DCE-MRI features and transcriptomic characteristics of BC. Tumor tissue was frozen and collected from 199 samples in the discovery cohort. Total RNA except for ribosomal RNA (rRNA) was extracted from tumor tissue using VAHTS Total RNA-seq (H/M/R) Library Prep Kit for Illumina in light of the manufacturer’s protocol, immediately frozen in liquid nitrogen, and stored at −80°C. RNA-seq libraries were constructed by Ovation human FFPE RNA-seq library systems (NuGEN Technologies, San Carlos, CA, USA) and sequenced on Illumina HiSeq X Ten platform (Illumina, San Diego, CA, USA) using paired-end 150-bp runs. Raw Illumina sequence reads were first processed by Trimmomatic (41) to remove sequencing adaptors and low-quality reads, using the following parameters: LEADING:3 TRAILING:5 SLIDINGWINDOW:4:15 MINLEN:60. RNA-seq reads were aligned to human genome 19 by STAR (42) and quantified by HTSeq-Count (43). The expression level of genes was quantified in the forms of both count data and normalized FPKM (fragments per kilobase of exon per million reads mapped). The sequencing coverage and quality statistics for each sample are summarized in **Supplementary File S2**. Expression values of 57,773 transcripts were determined, and the PAM50 intrinsic subtypes and risk scores of MammaPrint and Oncotype DX were calculated by using the R package genefu (44).

### Association analysis between dynamic contrast-enhanced–mri features and gene expression

We used Spearman’s rank correlation coefficients to calculate the linear relationship between each imaging feature and each gene expression level, resulting in two matrices (rows were imaging features, and columns were genes): one for the correlation coefficient r and the other for the *p*-value. Considering the imaging feature as a disease phenotype that was regulated by multiple genes, we corrected the *p*-value matrix by row using false discovery rates (FDRs) for the multiple comparisons. The imaging-associated genes were identified for the two cohorts under the criteria of both correlation coefficient r > 0.3 and q-value < 0.05. Gene enrichment analysis was conducted by Metascape (45) on the Kyoto Encyclopedia of Genes and Genomes (KEGG) pathways (*p*-value < 0.01).

### Imaging feature selection and imaging-based classifiers

We hoped to predict BC clinical receptors, IHC subtypes, PAM50 intrinsic subtypes, and prognostic gene sets using informative DCE-MRI features. Therefore, we needed to carry out the feature selection in the high-dimensional imaging features and further construct the machine learning classifiers. One-against-others strategy was used to build the binary classifiers in predicting the clinical receptors, subtypes, and prognostic gene sets. First, the discovery cohort was randomly divided into a training/validation set and a test set in a ratio of 7 to 3, and the validation cohort was used as an independent multi-institutional test set. Since high-throughput radiomics features were extracted, we performed an embedded feature selection procedure by using fivefold cross-validation and least absolute shrinkage and selection operator (LASSO) logistic regression (LR). Specifically, for each classification task, 100 times of the embedded feature selection procedure were applied to the training set, and features yielding the smallest classification error at each time were recorded as informative features. After that, the informative features for each classification task were obtained by counting the frequency.

Next, four machine learning algorithms, including elastic net regression (ENR), support vector machine (SVM), random forest (RF), and naïve Bayes (NB) were applied to establish classifiers by using feature forward search combined with grid search. The hyper-parameter alpha used to adjust the L1 and L2 penalties of ENR was set from 0 to 1 with a step of 0.1 and other parameters as default in training. The detailed hyper-parameters of SVM with polynomial kernel during training are as follows: the cost was from 1 to 15 with a step of 1, the degree was from 3 to 20 with a step of 1, and a dynamic gamma was used. If the number of model input imaging features *n_i_* was smaller than 20, the gamma was set from 0.01 to 2 × 1 ÷ *n_i_* with a step of 0.01; else, the gamma was set from 0.001 to 2 × 1 ÷ *n_i_* with a step of 0.001. For RF modeling, a seq of the parameter tree number from 100 to 2000 with a step of 50 was used and another parameter as default. Default parameters were used for NB classifier training. Performances were evaluated by the area under the receiver operating characteristic (ROC) curve (AUC), and accuracy (ACC). The cutoff of the ROC value was determined at the maximum Youden’s index. All of these were implemented in R 3.6.2. Then, a multi-classified neural network was also trained for PAM50 subtypes specifically. The activation function was set as ‘relu’, and the loss function was ‘categorical cross-entropy’. We used ‘Adam’ with default parameters as the iterator. Fivefold cross-validation was used to prevent overfitting. The model was constructed by Keras and TensorFlow in Python 3.5.2.

### Prognostic and statistical analyses

The Kaplan–Meier analysis with log-rank test was used to analyze the differences between DFS, overall survival (OS), or disease recurrence-free survival (DRFS). Univariate and multivariate Cox proportional risk regression analyses with the log-rank test were used to evaluate the risk of imaging feature on BC survival. Hazard ratio (HR) of risk genes or imaging features and 95% confidence interval (CI) were obtained by the Kaplan–Meier plotter or risk analysis (46).

Student’s t-test was used to compare the levels of DCE-MRI features in different groups. The correlation between gene expressions was calculated by Pearson’s correlation coefficient. The prognostic and statistical analyses were conducted in R 3.6.2.

## Results

### Clinical and transcriptomic characteristics of breast cancer samples

In **Table 1**, the clinical and transcriptomic characteristics of the two cohorts are listed. No significant difference was found in age, ER, PR, HER2, prognostic risk, or a pathological stage for the two cohorts. PAM50 subtypes differed between the two cohorts (Fisher’s exact test, *p*-value < 0.001). In the validation cohort, PAM50 luminal-A was dominant, while in the discovery cohort, five subtypes showed a roughly equal proportion. The luminal-A dominant bias was also found in TCGA-BRCA dataset compared with the discovery cohort (Pearson’s chi-squared test, *p*-value < 0.001). We thought the bias was probably due to ethnic differences, as the discovery cohort is composed entirely of the Chinese population. The results of substitutive typing based on IHC markers were significantly different from the intrinsic molecular subtypes in the discovery cohort (Pearson’s chi-square test, *p*-value < 0.001), suggesting that IHC-based subtyping still needed to be refined.

### Associations of dynamic contrast-enhanced–mri features and gene expression

**Figure 1:**
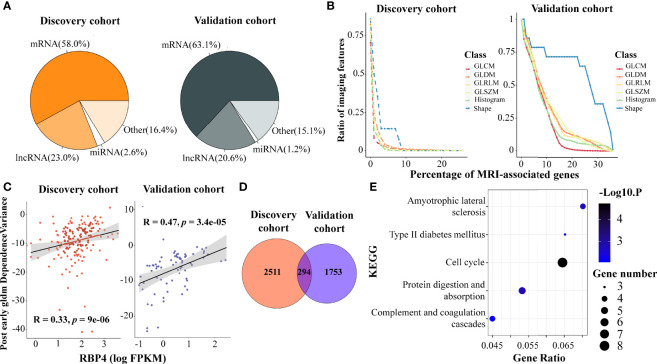
Association analysis of DCE-MRI features and BC transcriptomic characteristics. DCE-MRI features associated with molecules including mRNAs and non-coding RNAs in both cohorts **(A)**. Tumor shape features showed broader association with gene expression than other features. x-Axis represents the percentage of the number of genes, and y-axis denotes the percentage of the number of imaging features related to genes to the total number of features in this feature class. The point on the lower right corner of the curve means that there are fewer proportions of imaging features associated with more genes **(B)**. The expression of RAP4 was associated with the same imaging feature. x-Axis represents the log2-transformed value of FPKM gene expression, and y-axis denotes the imaging feature value **(C)**. A total of 294 MRI-associated genes overlapped in the two cohorts **(D)**, and five KEGG pathways including cell cycle were enriched in these overlapped genes **(E)**. DCE, dynamic contrast enhanced; BC, breast cancer; FPKM, fragments per kilobase of exon per million reads mapped; KEGG, Kyoto Encyclopedia of Genes and Genomes.

We identified 2,805 and 2,047 genes that correlated to DCE-MRI features in each cohort, under the criteria of both correlation coefficient *r* > 0.3 and q-value < 0.05. The proportion of MRI-associated genes was similar in the two cohorts (**Figure 1A**). Coding genes were more than half in the associated genes, and more than 20% of the associated genes were lncRNAs. It was interesting that most of the features were associated with a small number of genes, while only a few features related to a large number of genes, showing distribution in a power-law way (**Figures 1B**; **S2A–D**). Remarkably, the imaging features of tumor shape showed broader associations with gene expression. Nearly 14.2% of the shape features were linked to 7% of all the MRI-associated genes in the discovery cohort, and 35.7% were linked to 30% of all the MRI-associated genes in the validation cohort (**Figure 1B**). This indicated that shape features of imaging might represent more information on gene expression than other features.

Some genes showed a tight correlation with the imaging features. **Figure 1C** shows the correlation between gene RBP4 expression and gldm_DependenceVariance in the post-early MR images, with r = 0.33 and 0.47 in the two cohorts. RBP4 is proposed as an adipokine that links obesity and cancer. Recent research suggested that RBP4 could enhance the metastatic potential and increase the impairment of blood flow in BC tumors (47). Gene ADIPOQ could induce autophagic cell death in BC, and its expression is associated with some classical texture features from the pre-contrast images, such as pre_Uniformity (**Figure S2E**), pre_glcm_Contrast, and pre_glcm_Idm (48). We also found that some ncRNAs were associated with imaging features in BC. LINC00993 is a breast-specific lncRNA and acts as a tumor suppressor in BC (49). Feature like pre_LoG2_glszm_LargeAreaHighGrayLevelEmphasis (**Figure S2F**) could depict the expression of LINC00993. Moreover, LINC00993 was found to be related to the B-mode ultrasound phenotype of BC in literature (50).

### Dynamic contrast-enhanced–mri features reflected the activity of pathways in breast cancer

We performed functional analysis for the imaging feature-associated genes. In the discovery cohort, the associated 2,805 genes were enriched in 24 KEGG pathways (*p*-value < 0.01, **Figure S2G**), including ‘extracellular matrix (ECM) receptor interaction’, ‘pathways in cancer’, ‘complement and coagulation cascades’, ‘cytokine–cytokine receptor interaction’, ‘calcium signaling pathway’, ‘microRNAs in cancer’, ‘protein digestion and absorption’, and ‘hippo signaling pathway’. In the validation cohort, the 2,047 associated genes were enriched in 15 KEGG pathways (**Figure S2H**). Four pathways overlapped in both cohorts, including ‘calcium signaling pathway’, ‘protein digestion and absorption’, ‘regulation of lipolysis in adipocytes and glycine’, and ‘serine and threonine metabolism’.

We next identified 294 important MRI-associated genes that were present in both cohorts (**Figure 1D**), and 138 of them shared the same imaging gene pairs (**Supplementary File S3**). Particularly, five PAM50 marker genes, *MYBL2*, *MELK*, *EXO1*, *BCL2*, and *MKI67*, were included, suggesting that DCE-MRI features can indeed reflect the key molecular characteristics of BC. Importantly, five KEGG pathways were enriched in the overlapped genes (**Figure 1E**). Among them, the pathway ‘cell cycle’ obtained the most attention (*p*-value < 0.001), and eight imaging-associated genes (*CHEK1*, *TTK*, *CDC45*, *BUB1B*, *PLK1*, *E2F1*, *CDC20*, and *CDC25A*) enriched in this pathway. The results indicated a possibility of observing cancer-related pathways by DCE-MRI in a non-invasive way.

### A prognostic signature based on the eight imaging-associated genes

**Figure 2:**
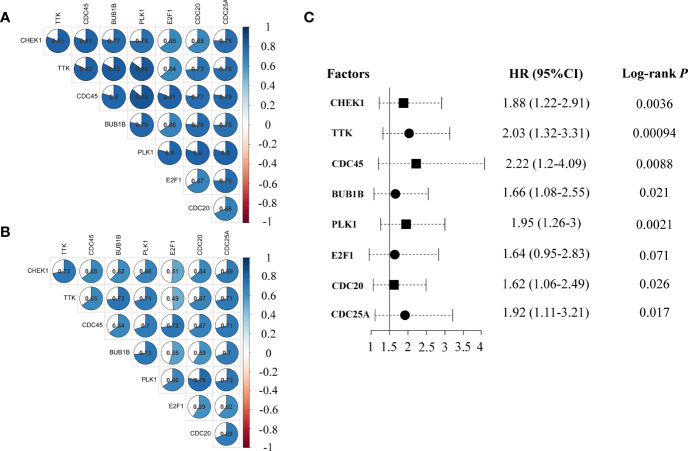
Co-expression and HRs of genes in BC-8mriG. The eight MRI-associated genes (BC-8mriG genes) were highly positively correlated in the discovery cohort (*n* = 174) and TCGA-BRCA dataset (*n* = 1090) **(A, B)**. Forest plot displays that BC-8mriG genes were all risk factors for BC **(C)**. HRs, hazard ratios; BC, breast cancer.

**Figure 3:**
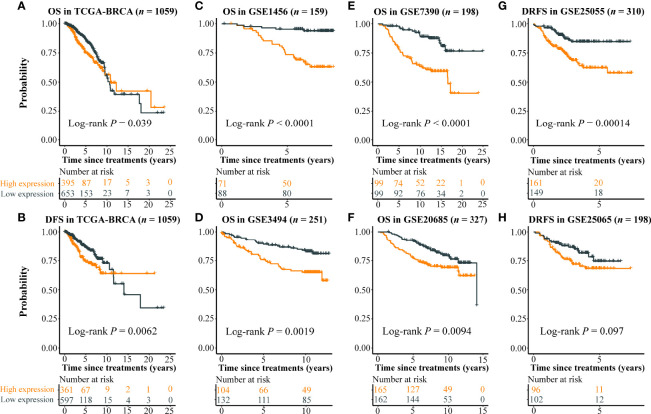
Prognostic ability of BC-8mriG. Patient stratification based on expression of BC-8mriG showed significantly different OS and DFS in TCGA-BRCA dataset **(A, B)**. The prognostic ability of BC-8mriG was validated in six external datasets by using Kaplan–Meier analysis **(C–H)**. OS, overall survival; DFS, disease-free survival.

The expression level of the eight imaging-associated genes, which were found in the cell cycle pathway, was highly correlated in each cohort (**Figures 2A, B**). High expression of these genes was a risk factor for DFS with HRs > 1.6 assessed by the Kaplan–Meier plotter (**Figure 2C**). With the use of the eight imaging-associated genes, a radiogenomics prognostic signature (named BC-8mriG) was developed to predict survival by calculating the average expression of the eight genes. The median of BC-8mriG values from a population was used as the cutoff to determine the high and low BC-8mriG expression patients in this population. In TCGA-BRCA dataset, significant differences in both OS and DFS were observed for the patients stratified by the median expression of BC-8mriG (**Figures 3A, B**, *p*-value = 0.039 and 0.0062, respectively). Such kinds of differences in OS or DFS were also found in the datasets GSE1456, GSE3494, GSE7390, GSE20685, and GSE25055 (**Figures 3C–G**), except for GSE25065 (**Figures 3H**). Although the results in TCGA-BRCA may be a slight discordance due to older age at onset (57.53 years in high BC-8mriG expression group and 60.05 years in low group) and longer follow-up, the survival patterns, namely, high and low expression of BC-8mriG corresponding to poor and favorable prognosis, were largely consistent in these datasets. This suggested the BC-8mriG was a reliable prognostic signature for BC. In comparison with other developed prognostic models (MammaPrint and Oncotype DX), BC-8mriG performed better (**Figure S3**).

### Imaging-based classifiers in predicting clinical receptors, subtypes, and prognostic gene sets

**Figure 4:**
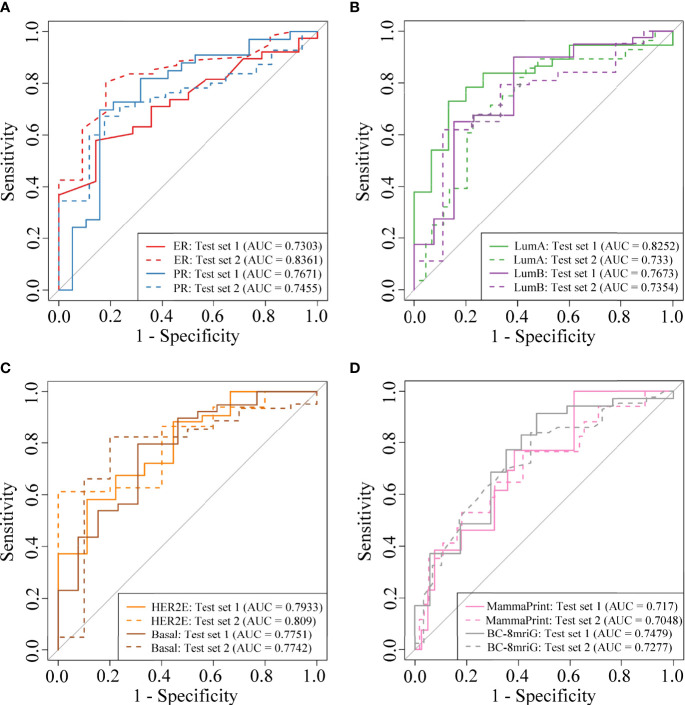
The model performance in two independent test sets. The ROC curves of the classifiers for ER status, PR status **(A)**, PAM50 luminal-A, luminal-B **(B)**, HER2-enriched, basal-like **(C)**, MammaPrint, and BC-8mriG **(D)** in the two cohorts. ROC, receiver operating characteristic; ER, estrogen receptor; PR, progesterone receptor; HER2, human epidermal growth factor receptor 2.

We established and validated the classifiers based on the DCE-MR imaging features to predict BC clinical receptors, subtypes, and prognostic gene sets. For each classification task, we used the imaging features of the best-preformed algorithm from the four machine learning methods as the final selected features. In **Supplementary File S4**, the informative imaging features obtained by using LASSO embedded LR algorithm, and the final selected imaging features were both listed for each classification task. Based on the selected imaging features, the classifiers for different tasks were established. SVM showed the best performance overall (**Figures S4A, B**), and the final hyper-parameters for the optimal classifiers were detailed in **Supplementary File S5**. In **Table 2**, we summarized the performance of the classifiers, including the number of the selected features, AUC, and accuracy in the two independent test sets. Our models showed better performance compared with other studies of the prediction for receptors status and clinical subtypes (**Table S1**). The AUCs for ER status were 0.7303 and 0.8361, and the AUCs for PR status were 0.7671 and 0.7455 (**Figure 4A**). Importantly, we established the PAM50 molecular subtype classifiers based on DCE-MRI features for the first time to our knowledge, and the models performed well in the external test set, with AUCs of 0.733, 0.7354, 0.809, and 0.7742 for luminal-A, luminal-B, HER2-enriched, and basal-like, respectively (**Figures 4B, C**). The results demonstrated the feasibility of predicting the PAM50 subtypes of BC only based on quantitative imaging features. We also built risk degree prediction models for MammaPrint and BC-8mriG and obtained the AUCs of 0.7048 and 0.7277 in the test sets (**Figure 4D**).

**Table 2:** Predictive performance of classifiers based on DCE-MRI features.

Classification tasks	Features selected	Independent internal test set (*n* = 52)		Independent external test set (*n* = 72)	
		AUC	ACC	AUC	ACC
ER (+ vs −)	95	0.7303	0.6538	0.8361	0.8056
PR (+ vs −)	19	0.7671	0.75	0.7455	0.6667
HER2 (+ vs −)	47	0.7539	0.75	0.61	0.5686
Ki67 (high vs low)	16	0.8958	0.7885	NA	NA
IHC: LumA vs Not-LumA	6	0.7855	0.7692	NA	NA
IHC: LumB vs Not-LumB	3	0.7061	0.7115	NA	NA
IHC: HER2p vs Not-HER2p	8	0.9042	0.8302	NA	NA
IHC: TN vs Not-TN	51	0.8295	0.7115	NA	NA
PAM50: LumA vs Not-LumA	20	0.8252	0.7692	0.733	0.7361
PAM50: LumB vs Not-LumB	5	0.7673	0.8302	0.7354	0.6528
PAM50: HER2E vs Not-HER2E	2	0.7933	0.6346	0.809	0.6389
PAM50: Basal vs Not-Basal	2	0.7751	0.7692	0.7742	0.8194
MammaPrint	6	0.717	0.6538	0.7048	0.75
BC-8mriG	29	0.7479	0.7885	0.7277	0.7222

Models were established for predicting clinical receptors, subtypes, and prognostic gene sets and validated in both independent test sets. Important model evaluation indicators were also calculated.

+, positive; −, negative; LumA, luminal-A; LumB, luminal-B; HER2p, HER2 positive; TN, triple-negative; HER2E, HER2-enriched; Basal, basal-like; AUC, area under the receiver operating characteristic curve; ACC, accuracy; NA, not available; ER, estrogen receptor; PR, progesterone receptor; IHC, immunohistochemistry.

**Figure 5:**
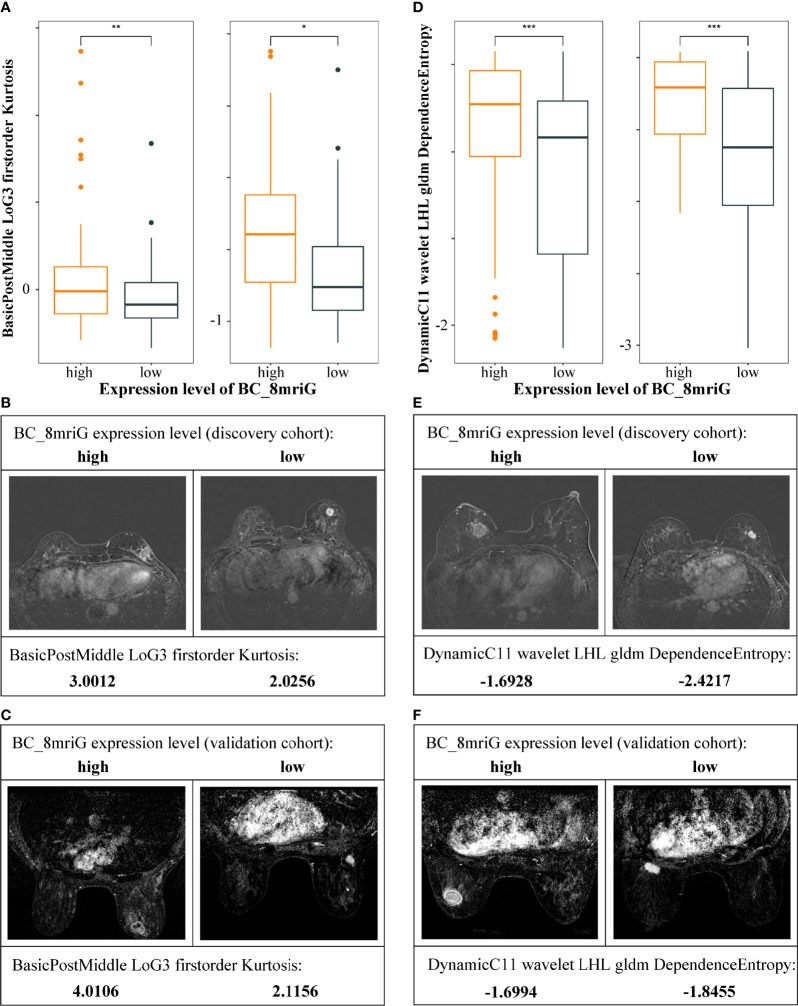
Heterogeneity of DCE-MRI features in different BC-8mriG expression patients. DCE-MRI features BIF1 **(A–C)** and BIF2 **(D–F)** were significantly different in samples with high and low expression of BC-8mriG in both discovery and validation cohorts (*, **, and ***: *p*-values < 0.05, 0.01, and 0.001, respectively). DCE, dynamic contrast enhanced.

For the classifier of BC-8mriG, 29 important DCE-MRI features (detailed in **Supplementary File S4**) were selected and used. We further tested the classifying capacity of the 29 imaging features in the patients with high and low gene expression of BC-8mriG. The result showed that two imaging features, BIF1 (BasicPostMiddle LoG3 firstorder Kurtosis) and BIF2 (DynamicC11 wavelet LHL gldm DependenceEntropy), exhibited significantly different levels between patients with high and low expression of the eight genes in both discovery and validation cohorts (**Figures 5A, D**). In both cohorts, the high level of BIF1 was found in BC-8mriG high expression patients, while the low level of BIF1 corresponded to BC-8mriG low expression (**Figures 5B, C**). A similar result was observed for DCE-MRI feature BIF2 (**Figures 5E, F**).

Furthermore, a multi-classified neural network model was constructed to classify PAM50 molecular subtypes of BC. Some of the informative imaging features overlapped in the four binary PAM50 classification tasks (**Figure S4C**). We selected 337 imaging features to build the multi-classified model for the prediction of PAM50 subtypes. The fivefold cross-validation method was used to determine the optimal epoch in the discovery cohort, and eventually, our model was tested in the independent test set. The performance of evaluation metrics for our multi-classified model is shown in **Figures S4D–F**. The final model was determined when the highest AUC value was obtained for the validation set (AUC = 0.803), and the multi-classified AUC in the test set was 0.6622.

### Prognostic analysis with dynamic contrast-enhanced–mri features

**Figure 6:**
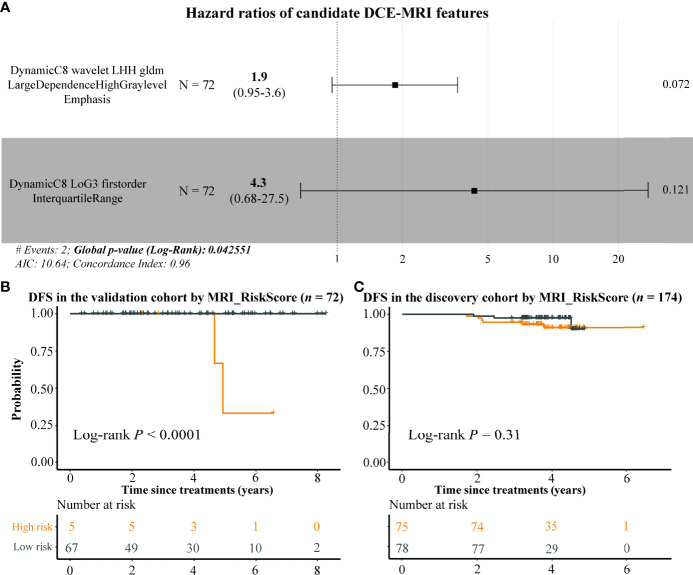
The potential prognostic ability of DCE-MRI features. Forest plot by using multivariate Cox regression analysis indicates that BIF3 and BIF4 were risk factors of BC **(A)**, and the Kaplan–Meier plots display the DFS differences between high and low MRI risk score BC samples in the validation and discovery cohorts **(B, C)**. DCE, dynamic contrast enhanced; BC, breast cancer; DFS, disease-free survival.

In order to extend the clinical application, we attempted to construct a prognostic model only using DCE-MRI features. Due to the short time of follow-up data in the discovery cohort, we assessed the BC prognosis of the 29 DCE-MRI features in the validation cohort by using univariate Cox proportional risk regression analysis first, resulting in only one imaging feature that was related to the DFS of BC significantly (BIF3 (DynamicC8 wavelet LHH gldm LargeDependenceHighGrayLevelEmphasis), *p*-value = 0.0214). Therefore, we selected the top 2 imaging features with the smallest *p*-value as candidate risk features and calculated their HRs by using multivariate Cox analysis. The HRs of imaging features BIF3 and BIF4 (DynamicC8 LoG3 firstorder InterquartileRange) were 1.9 and 4.3, respectively, indicating that they were risk factors for DFS with a *p*-value of 0.0426 (**Figure 6A**). Using the two DCE-MRI features, we constructed a radiomics signature (named as MRI_RiskScore) for BC. In the validation cohort, BC samples were stratified by the mean of MRI_RiskScore, and highly significant differences in DFS were observed between high- and low-risk samples (**Figure 6B**, log-rank *p*-value < 0.0001). Although MRI_RiskScore showed less prognostic prediction in the discovery cohort, BC patients with high scores still tended to have a bad clinical outcome (**Figure 6C**). Overall, the results exhibited that the two DCE-MRI features can partially predict BC prognosis.

## Discussion

In this study, 246 BC samples were organized from a local institution and a public database for radiogenomics analysis. The discovery cohort (*n* = 174) was a Chinese population cohort, and the validation cohort (*n* = 72) was a public dataset. In addition, 1,443 BC cases were also collected from the GEO database. Our results indicated that gene expression broadly correlated with DCE-MRI features. Consistent with previous findings, the KEGG pathway cell cycle was also found to be closely associated with DCE-MRI features (*p*-value < 0.001). Based on the relationship, we developed a prognostic signature (BC-8mriG) and validated it with good prognostic power in multiple datasets (*p*-value < 0.01). We further developed and validated classifiers for IHC receptors, subtypes, and gene sets only based on the imaging features. Particularly, as we know we were the first study to predict PAM50 subtypes based on DCE-MRI features directly. Furthermore, our results suggested that DCE-MRI features might be an independent predictor of BC outcome. In general, we analyzed the association of DCE-MRI features with expression characteristics, molecular pathways, clinical receptors, subtypes, and prognosis, providing a non-invasive way of understanding BC.

PAM50 intrinsic subtype is the most important and widely used molecular subtyping system of BC. Each subtype has a distinct gene expression pattern, and diverse behavior in molecular mechanism, histological characteristic, clinical presentation, and treatment response. In our work, we found that the distribution of PAM50 subtypes in the Chinese population (Chinese Han) was different from that in other races (two-sided Pearson’s chi-squared test, *p*-value < 0.001). Previous studies reported that BC patients in diverse populations had distinct prevalence and mortality rates for different subtypes (51–54). For example, the prevalence of luminal-A BC in Chinese Han was remarkably lower than that in Caucasian whites (51, 53), blacks with luminal-A or luminal-B tumors were more likely to die of BC, and Asians usually had a lower mortality rate than whites (52). A recent large cohort study including 6,652 BC patients showed that patients of different races had different genomic characteristics, such as TP53 variations occurred more often in blacks than whites or Asians, which might be a potentially important factor in the racial heterogeneity of the PAM50 intrinsic molecular subtypes of BC (55). Racial disparities of BC remain a persistent challenge in clinical practice, particularly for some therapeutic strategies based on targetable genes, which require more focused research in the future.

Radiogenomics is a promising approach to realizing precision medicine by using non-invasive imaging technology to monitor the molecular behavior of the tumor, as the latest studies reported (56–60). For instance, the tumor mutational burden risk can be predicted in both primary and liver-metastatic colorectal cancer (AUCs: 0.732 and 0.812) by using radiogenomics analysis based on computed tomography (CT) images (57). Radiomics features from positron emission tomography (PET) imaging of ^18^F-fluorodeoxyglucose (FDG) markedly related to the activation and alteration of mTOR pathway genes in hepatocellular carcinoma (58), and similar results were also reported in BC that some immune-related pathways were associated with FDG-PET features, such as flux constants and static uptake (59), and some researchers also aimed to predict Ki-67 status from multiparametric MRI images (AUC: 0.79) in BC (60). In addition, integration of radiomics and genomic features is also a promising area, such as the radiogenomics model (AUC: 0.87) showed much better performance than the radiomics-only models (AUCs: 0.71 and 0.73) in the prediction of pathological complete response of TNBC (61). In this work, a number of important genes and pathways associated with BC were found to be associated with imaging features. For example, MYBL2 expression was correlated with 42 imaging features such as LoG3_glcm_Idm and LoG3_glcm_Contrast of post-middle and post-later MR images in both cohorts, and a high expression of MYBL2 usually means BC metastasis, worse DRFS, and shorter OS (62). Interestingly, we observed that several adipocyte-related genes such as *LEP* and *FABP4* displayed plenty of associations with imaging features. *LEP* is a multifunctional hormone secreted from adipocytes, linking obesity to BC, and may play important roles in BC development (63). *FABP4* is also a key adipokine produced by adipocytes and is mainly involved in the transport of fatty acids. Recent research demonstrated that *FABP4* can promote obesity-associated BC development and may be a novel player linking obesity and BC risk (64). This result suggested that MR imaging features could capture the molecular characteristics of both intratumoral and microenvironment, extending the potential application scenarios of radiogenomics.

Previous studies indicated that imaging features could reflect the expression activities of gene sets that have specific functions (15, 18–20). In our work, we observed MRI-associated genes enriched in some KEGG pathways, especially in the cell cycle. Cell cycle deregulation is regarded as a hallmark of malignant that enables limitless cell division of tumor cells and is likely to represent cell proliferation and can be used for prognostication. Notably, consistent with our results, some researchers also found an association between imaging features and the deregulation of the cell cycle (19, 20, 65). Some pathways involved in the process of extracellular material exchange activities, such as extracellular matrix (ECM) receptor interaction, protein digestion and absorption, cytokine–cytokine receptor interaction, and cell adhesion molecules, were also been found to be significantly associated with imaging features in our cohort. Similar results were reported in other studies as well, which reflected the reliability of our results and the potential clinical value of quantitative imaging features in characterizing the proliferation and metabolism of breast tumor cells.

Prognostic analysis based on imaging features has always been a research hotspot in radiomics and radiogenomics. In this work, we identified eight enriched MRI-associated genes including *CHEK1*, *TTK*, *CDC45*, *BUB1B*, *PLK1*, *E2F1*, *CDC20*, and *CDC25A* from the cell cycle and further discovered that these genes had the ability to predict the prognosis of BC in TCGA-BRCA dataset. Our results revealed that these genes were not only risk factors for BC but also related to DCE-MRI features. We further developed the BC-8mriG as a prognosis indicator of BC, and higher expression of BC-8mriG indicated a worse outcome. Moreover, BC-8mriG displayed better prognostic capabilities in multiple datasets compared with MammaPrint and Oncotype DX gene assays in this study. A machine learning model was also built for predicting the expression level of BC-8mriG based on imaging features, providing a radiogenomics approach to analyzing prognosis non-invasively. In addition, we directly assessed the prognostic ability of important DCE-MRI features and noted their clinical value as independent prognostic indicators.

Molecular heterogeneity analysis greatly improved the treatment outcome of BC. Many studies have tried to establish machine learning models based on imaging features to predict clinical receptors and IHC subtypes (26–30). However, no similar research published the PAM50 subtypes. Therefore, not only receptor status and IHC subtypes but also PAM50 subtypes were modeled in this work. The performance of machine learning models was evaluated in two independent datasets, and our results were comparable to other studies. Four different machine learning algorithms were used, with SVM performing best overall. This may be due to SVM, which is known as a powerful tool for using different kernels for classification and regression analysis in a small-size dataset, and especially SVM can model the non-linear decision boundary and is robust against overfitting during training. To our knowledge, this is the first study to use DCE-MRI features to predict the PAM50 subtypes and to validate them in an external dataset, which may provide support for BC diagnosis.

Compared with previous radiogenomics studies, although our work overcame a few shortcomings, it still had some limitations (56, 66, 67). First, we used two radiogenomics cohorts of BC: one was a local single-center dataset for discovery (*n* = 174) and another was a public multi-center dataset for validation (*n* = 72). Although our data size had increased and used an independent validation dataset, the generalization ability of revealed associations and prediction models still needed to be verified in a larger multi-center cohort. In addition, the performance of our models still needed to be improved in the future, especially for the prediction of PAM50 intrinsic subtypes, and there was also a requirement to develop a well-performed PAM50 multi-classified predictor. The establishment of robust and reproducible radiomics-genomics associations was an important bottleneck hindering the clinical application of radiogenomics. Second, although we made some efforts to reduce the systematic bias in imaging and sequencing data, such as N4 bias correction for 3T-MRI data and voxel normalization, systemic differences in this work still existed as the images of the validation cohort were generated by different manufacturers in 1.5 T, which might result in the relatively poor performance of models in the validation dataset. Third, we used manual segmentation for regions of interest, which was a time-consuming and labor-intensive approach, and the high-throughput imaging features were abstract, leading to the interpretability lack for the majority of features. Deep learning-based radiogenomics analysis may be a promising way in future works (68–70). Furthermore, although we found some genes with prognostic value based on the association of imaging features and gene expression profiles, the direct prognostic prediction of imaging features did not perform very well. We are looking forward to analyzing the prognostic value of radiomics features in BC by using new and larger datasets in the next step, and we also hope to expand the use of radiomics features in future work, particularly in the assessment treatment responses.

## Conclusions

In this work, we conducted BC radiogenomics analysis based on DCE-MRI and RNA-seq data of 246 patients from multiple centers. Reliable associations between DCE-MRI features and gene expression profiles were identified and validated, and the cell cycle pathway was found to be the most related to radiomics features. Based on the associations, a radiogenomics prognostic signature including eight genes was developed and performed well in multiple datasets. By using machine learning analysis, we further established radiomics-based models to predict the clinical receptors, PAM50 subtypes, and prognostic signatures in BC. Despite the good performance of our models, there is still a need to improve model performance and generalization to meet clinical needs. In addition, our results suggested that DCE-MRI features were potential biomarkers of BC outcomes, which still need to be further revealed in future works.

## Data availability statement

The datasets presented in this study can be found in online repositories. The names of the repository/repositories and accession number(s) can be found below: https://ngdc.cncb.ac.cn/gsa-human/, HRA001100.

## Ethics statement

The studies involving human participants were reviewed and approved by the ethical committee of the First Affiliated Hospital of Nanjing Medical University. The patients/participants provided their written informed consent to participate in this study.

## Author contributions

HoL, XL, YL, and XS conceptualized and designed this study. YZ, TX, and XL collected samples, and the pathological review was performed by YZ and XY. FL, YB, and WG completed the RNA sequencing. RNA-seq data analysis was performed by ZH, WM, and HuL. MR images were collected by ZD, and tumor semi-automatic segmentation was completed by WM, YG, and YZ. WM completed the radiogenomics analysis, including imaging feature extraction, statistical analysis, and machine learning model construction. SY, RZ, WZ, and HaL helped in the construction of machine learning models. The draft manuscript was developed by WM, HL, JD, and XS. All authors reviewed the draft and provided comments, contributing to the final version of the manuscript. The work reported in the paper has been performed by the authors unless clearly specified in the text.

## Funding

This work was funded by the National Key R&D Program of China (2018YFC1314900, 2018YFC1314902), Bethune Charitable Foundation (G-X-2019-0101-12), National Natural Science Foundation of China (61972084, 61871121), and Key Research & Development Program of Jiangsu Province (BE2016002-3).

## Acknowledgments

We thank Wenjing Cui from the Department of Radiology, Jiangsu Province Hospital of Chinese Medicine, for her contribution to tumor segmentation, and Jiansheng Wu from the School of Geography and Biological Information, Nanjing University of Posts and Telecommunications, for his help in the construction of neural network model.

## Conflict of interest

The authors declare that the research was conducted in the absence of any commercial or financial relationships that could be construed as a potential conflict of interest.

## Publisher’s note

All claims expressed in this article are solely those of the authors and do not necessarily represent those of their affiliated organizations, or those of the publisher, the editors and the reviewers. Any product that may be evaluated in this article, or claim that may be made by its manufacturer, is not guaranteed or endorsed by the publisher.
