# Supplementary material for: Radiogenomics analysis reveals the associations of dynamic contrast-enhanced–MRI features with gene expression characteristics, PAM50 subtypes, and prognosis of breast cancer
Source: Front Oncol. 2022 Jul 28;12:943326. doi: 10.3389/fonc.2022.943326 (PMC9366134; doi:10.3389/fonc.2022.943326)
Supplement: Supplementary file 1 [file DataSheet_1.docx]

**Supplementary File S1** for

**Radiogenomics analysis reveals the associations of DCE-MRI features with gene expression characteristics, PAM50 subtypes and prognosis of breast cancer**

Wenlong Ming, Yanhui Zhu, Yunfei Bai, Wanjun Gu, Fuyu Li, Zixi Hu, Tiansong Xia, Zuolei Dai, Xiafei Yu, Huamei Li, Yu Gu, Shaoxun Yuan, Rongxin Zhang, Haitao Li, Wenyong Zhu, Jianing Ding, Xiao Sun, Yun Liu^*^, Hongde Liu^*^, Xiaoan Liu^*^

^*^Co-corresponding authors’ emails: [liuyun@njmu.edu.cn](mailto:liuyun@njmu.edu.cn); [liuhongde@seu.edu.cn](mailto:liuhongde@seu.edu.cn); [liuxiaoan@126.com](mailto:liuxiaoan@126.com)

**The file includes:**

**Supplementary Methods.**

**Fig. S1.** Flowchart describing the inclusion criteria and analysis process.

**Fig. S2****.** Association between DCE-MRI features and transcriptomic characteristics of BC.

**Fig. S3.** Prognostic ability of MammaPrint and Oncotype DX gene assays.

**Fig. S4.** Performance comparison of classifiers and multi-classified neural network model for PAM50 subtypes.

**Table S1.** Performance comparison of our classification models with other studies.

**Table S2.** Summary of 1,046 basic radiomics features.

# Supplementary Methods

**Detail of quantitative DCE-MR imaging features**

Totally, 15,494 high-throughput quantitative imaging features were calculated for each case, including 14 shape features, 4,128 basic statistic and filtered features (Basic features), and 11,352 dynamic features (Dynamic features). The 4,128 basic statistic and filtered features (1,032*4 = 4,128) were obtained by calculating 1,032 basic imaging features (86 histogram and texture features of original images, 258 Laplacian of Gaussian (LoG) filter features, and 688 wavelet features, according to the IBSI Reporting Guidelines) of one pre-contrast and three post-contrast MR images. The 11,352 dynamic features (1,032*11 = 11,352) were consisted of two parts; one was the absolute variation of the 1,032 basic imaging features for early, middle and late stages of post-contrast MRI compared to pre-contrast MRI (DynamicC1 to DynamicC3). The latter was eight statistical characteristics of 1,032 basic imaging features in the four stages, including maximum, minimum, mean, median, variance, standard deviation, skewness and kurtosis (DynamicC4 to DynamicC11). **Table S2** detailed the 14 shape and 1,032 basic imaging features.

# **Supplementary Figu**res


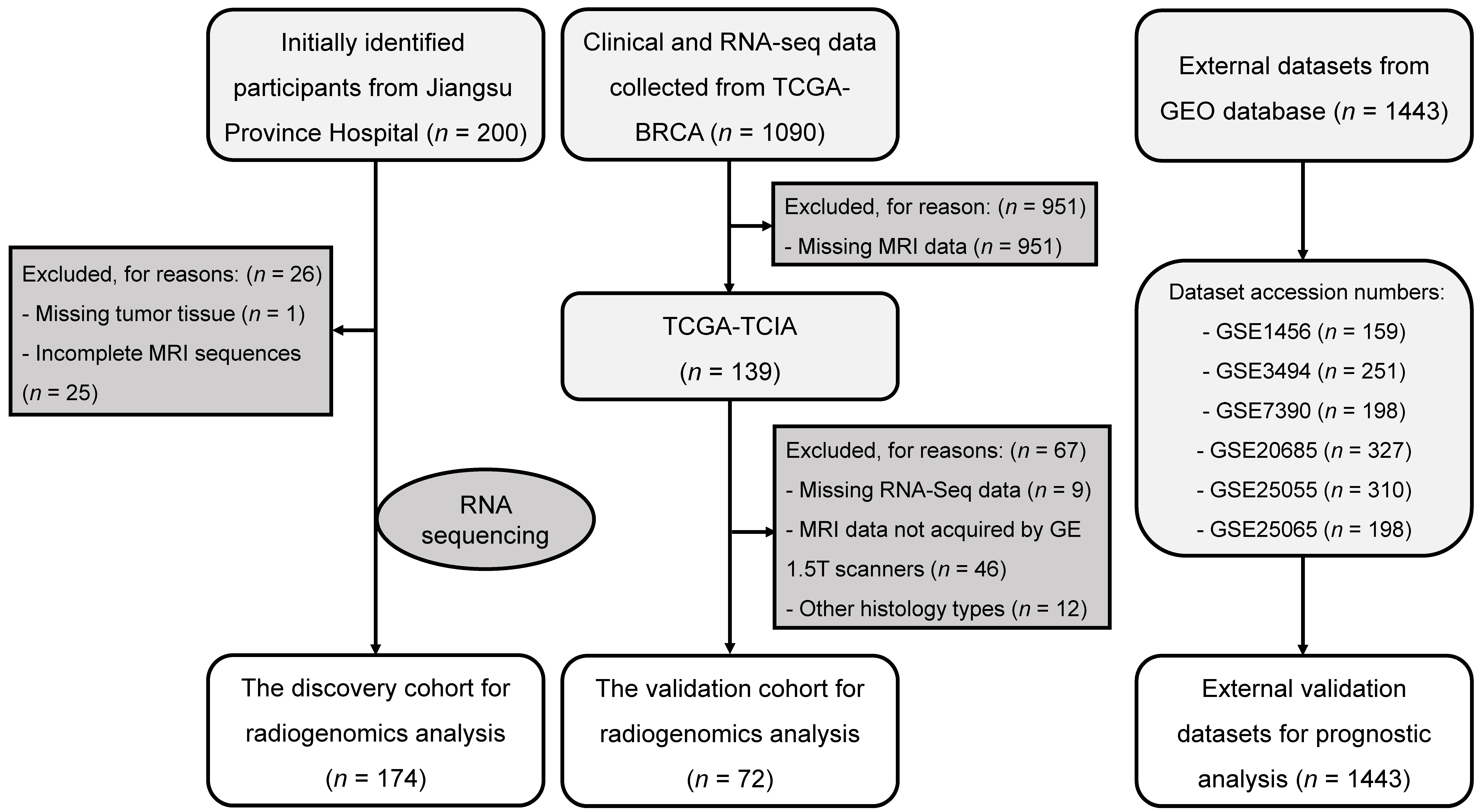


**Figure S1. Flowchart describing the inclusion criteria and analysis process.** The discovery cohort was a Chinese population dataset and the validation cohort was a public dataset from TCGA and TCIA database. 246 samples were enrolled in the radiogenomics analysis, and six external datasets including 1,443 samples were also retrieved from GEO to assess prognosis.


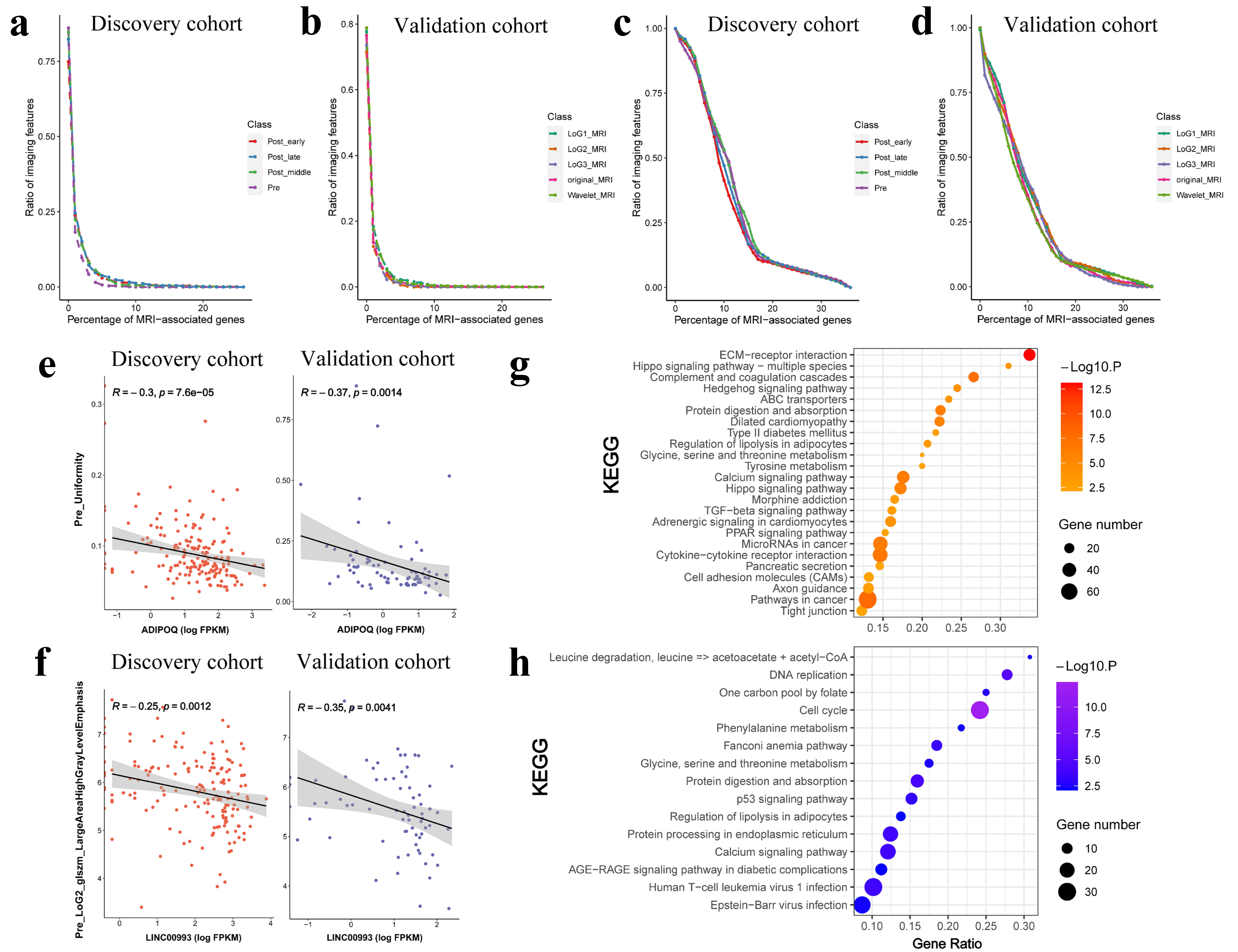


**Figure S2. Association between DCE-MRI features and transcriptomic characteristics of BC.** Imaging features extracted from contrast enhanced MR images (**a and c**), original and filtered MR images (**b and d**) correlated with different numbers of genes in the discovery and validation cohorts. X-axis represents the percentage of the number of genes, and y-axis denotes the percentage of the number of imaging features related to genes to the total number of imaging features in this feature class. The point on the lower right corner of the curve means that there are fewer proportions of imaging features associated with more genes (**a-d**). ADIPOQ and lncRNA LINC00993 were associated with different imaging features in both cohorts. X-axis represents the log2-transformed value of FPKM gene expression, and y-axis denotes the imaging feature value (**e and f**). MRI-associated genes enriched in 24 and 15 KEGG pathways in the two cohorts respectively (**g and h**).

**
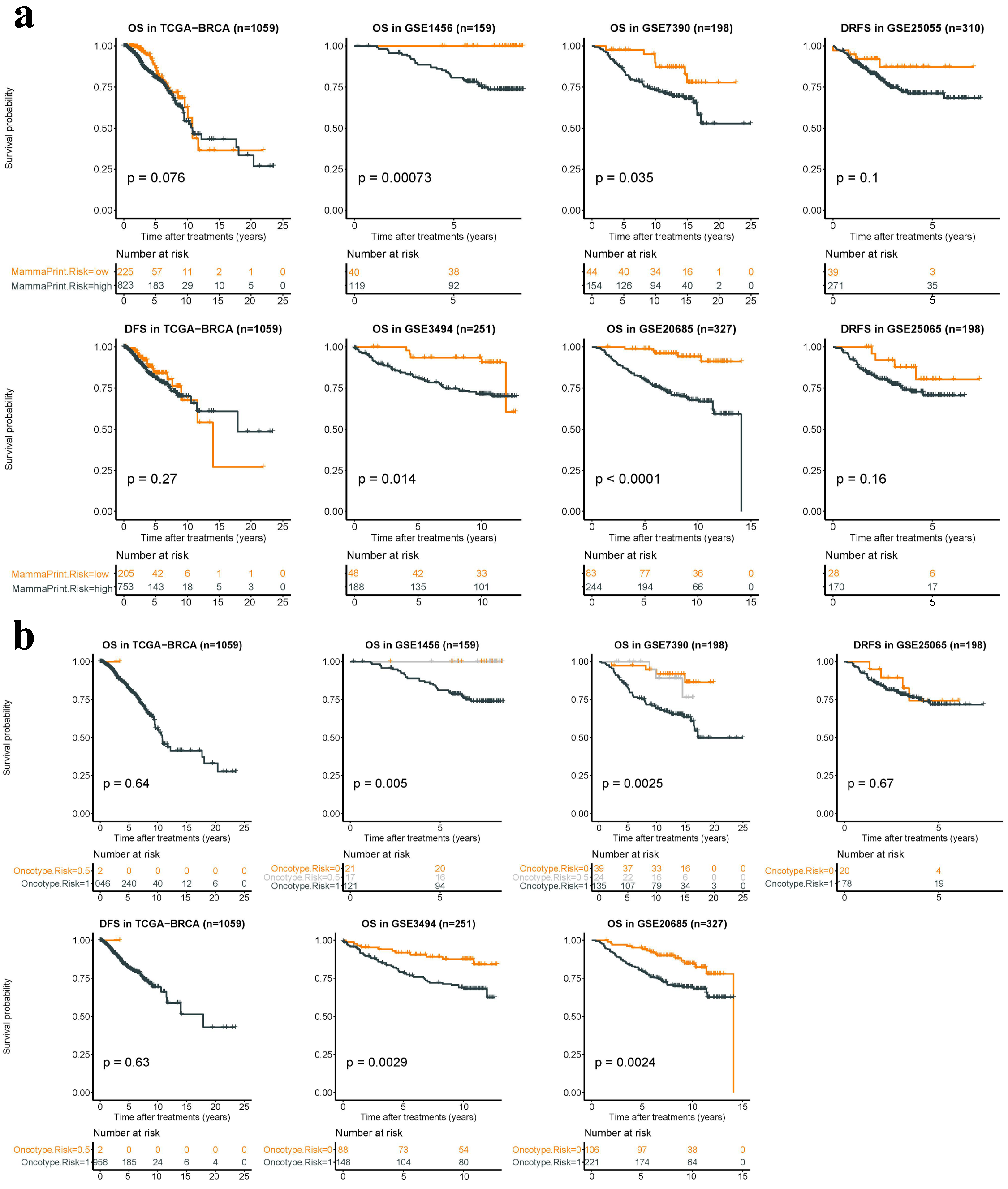
**

**Figure S3. Prognostic ability of MammaPrint and Oncotype gene assays.** Risk scores were obtained using MammaPrint and Oncotype gene assays, and BC patients were then stratified by risk scores. The outcomes of patients at different risk were assessed in multiple datasets, and MammaPrint gene assay showed a not bad prognostic capacity by classifying patients into high-risk and low-risk groups (**a**). Oncotype method evaluated patients into high-risk (risk score = 1), intermediate-risk (risk score = 0.5) and low-risk (risk score = 0), and had some prognostic ability (**b**).

**
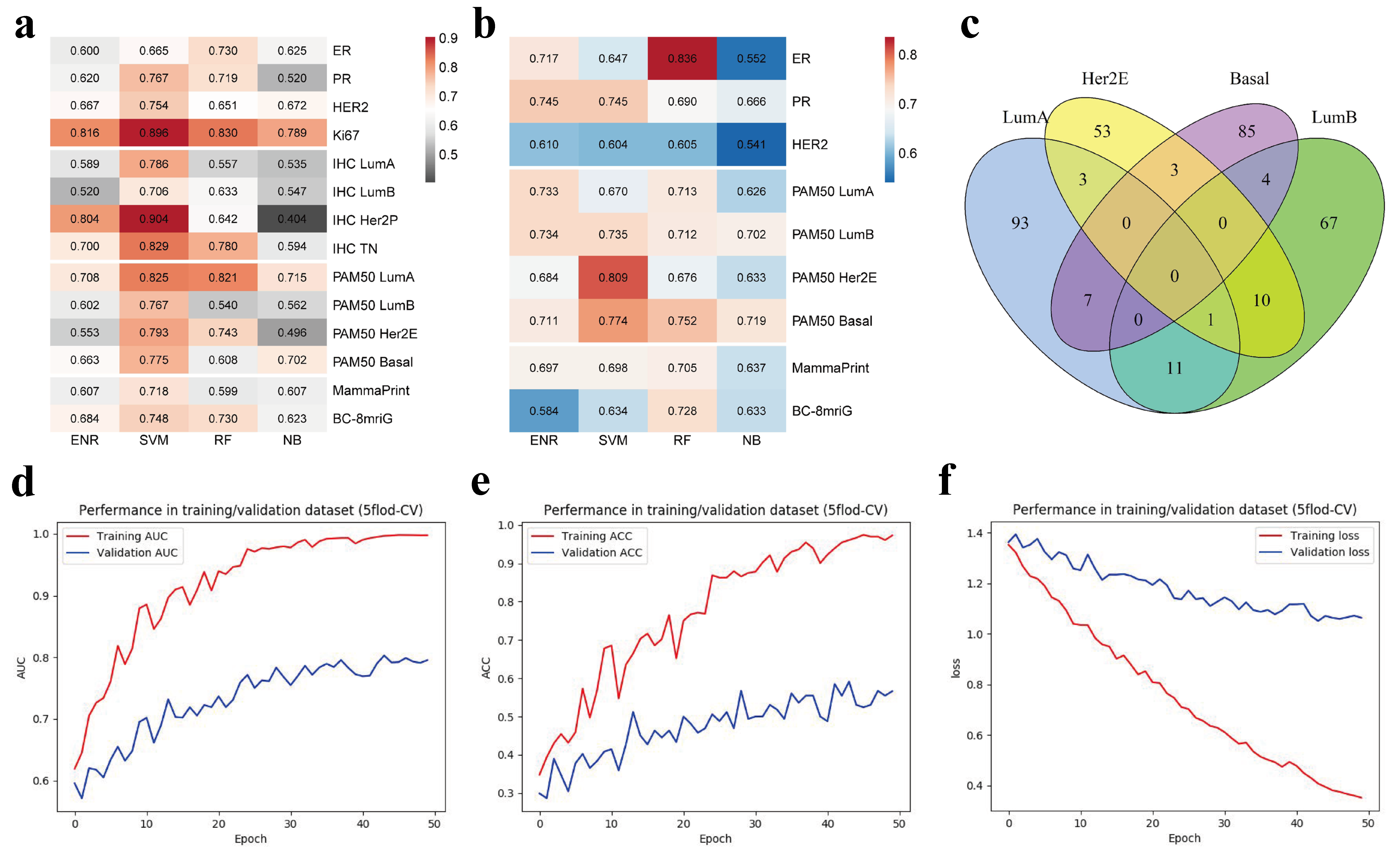
**

**Figure S4. Performance comparison of classifiers and multi-classified neural network model for PAM50 subtypes.** The best AUC performance of machine-learning models for each classification task in the test set from the discovery cohort (**a**) and the test set from the validation cohort (**b**). Venn plot of imaging features in different PAM50 classification tasks (**c**). The performance of key evaluation metrics of model in the discovery and validation cohorts, including AUC, accuracy (ACC), and function loss (**d-f**). LumA, luminal-A; LumB, luminal-B; HER2p, HER2 positive; TN, triple-negative; HER2E, HER2-Enriched; Basal, basal-like.

# Supplementary Tables

**Table S1. Performance comparison of our classification models with other studies.** For IHC receptors status and clinical subtype prediction tasks, our models showed better performance compared with other studies. Importantly, PAM50 molecular subtype prediction models performed well in the external test set.

| **Classification tasks** | **Our results** | | **Agner SC, et al. (28)** | **Wang J, et al. (29)** | **Li H, et al. (25)** | **Li H, et al. (27)** | **Wu J, et al. (30)** | **Saha A, et al. (26)** |
| --- | --- | --- | --- | --- | --- | --- | --- | --- |
|  | **Internal test set** | **External test set** |  |  |  |  |  |  |
| ER (+ vs -) | 0.7303 | 0.8361 | NA | NA | 0.89 | NA | NA | 0.649 |
| PR (+ vs -) | 0.7671 | 0.7455 | NA | NA | 0.69 | NA | NA | 0.622 |
| HER2 (+ vs -) | 0.7539 | 0.61 | NA | NA | 0.65 | NA | NA | 0.500 |
| Ki67 (high vs low) | 0.8958 | NA | NA | NA | NA | NA | NA | 0.624 |
| IHC: LumA vs Not-LumA | 0.7855 | NA | NA | NA | NA | NA | 0.73 | 0.697 |
| IHC: LumB vs Not-LumB | 0.7061 | NA | NA | NA | NA | NA | 0.69 | 0.566 |
| IHC: HER2p vs Not-HER2p | 0.9042 | NA | NA | NA | NA | NA | NA | 0.633 |
| IHC: TN vs Not-TN | 0.8295 | NA | 0.73 | 0.782 | 0.67 | NA | 0.79 | 0.654 |
| PAM50: LumA vs Not-LumA | 0.8252 | 0.733 | NA | NA | NA | NA | NA | NA |
| PAM50: LumB vs Not-LumB | 0.7673 | 0.7354 | NA | NA | NA | NA | NA | NA |
| PAM50: HER2E vs Not-HER2E | 0.7933 | 0.809 | NA | NA | NA | NA | NA | NA |
| PAM50: Basal vs Not-Basal | 0.7751 | 0.7742 | NA | NA | NA | NA | NA | NA |
| MammaPrint | 0.717 | 0.7048 | NA | NA | NA | 0.88 | NA | NA |

Note: unless otherwise indicated, data are the AUC values of classification models. +, positive; -, negative; LumA, luminal-A; LumB, luminal-B; HER2p, HER2 positive; TN, triple-negative; HER2E, HER2-Enriched; Basal, basal-like; NA, not available.

**Table S2. Summary of 1,046 basic radiomics features.**

| **Feature classes** | ***n* of features** | **3 representative features** |
| --- | --- | --- |
| Shape  First order  GLCM  GLSZM  GLRLM  GLDM  Log-sigma  Wavelet  Total | 14  18  22  16  16  14  258  688  1,046 | Mesh Volume, Surface Area, Sphericity, …  Energy, Entropy, Mean, …  Autocorrelation, Joint Average, Contrast, …  Small Area Emphasis, Large Area Emphasis, Gray Level Non-Uniformity, …  Short Run Emphasis, Long Run Emphasis, Gray Level Non-Uniformity, …  Small Dependence Emphasis, Large Dependence Emphasis, Gray Level Non-Uniformity, …  Log-sigma 1-mm, 2-mm, 3-mm-related features, …  Wavelet-HHH, LLL, HHL related features, … |

Note: *n* = number, GLCM = Gray Level Co-occurrence Matrix, GLSZM = Gray Level Size Zone Matrix, GLRLM = Gray Level Run Length Matrix, GLDM = Gray Level Dependence Matrix.
